# Overcoming the barriers of teaching physical examination at the bedside: more than just curriculum design

**DOI:** 10.1186/s12909-018-1403-z

**Published:** 2018-12-11

**Authors:** Melissa Rousseau, Karen D. Könings, Claire Touchie

**Affiliations:** 1 Department of Medicine, The Ottawa Hospital, University of Ottawa, 501 Smyth Road CPCR L2135, Box 209, Ontario, Ottawa K1H 8L6 Canada; 2 Department of Educational Development and Research, Faculty of Health, Medicine and Life Sciences, Maastricht University, the Netherlands, Universiteitssingel 60, Room M 5.08, 6229 ER Maastricht, The Netherlands; 3 Department of Medicine, The Ottawa Hospital, University of Ottawa and the Ottawa Hospital Research Institute, 501 Smyth Road CPCR L2135, Box 209, Ontario, Ottawa K1H 8L6 Canada

**Keywords:** Curriculum, Medical education – Clinical skills training, Qualitative methods, Physical examination, Bedside

## Abstract

**Background:**

Physicians in training must achieve a high degree of proficiency in performing physical examinations and must strive to become experts in the field. Concerns are emerging about physicians’ abilities to perform these basic skills, essential for clinical decision making. Learning at the bedside has the potential to support skill acquisition through deliberate practice. Previous skills improvement programs, targeted at teaching physical examinations, have been successful at increasing the frequency of performing and teaching physical examinations. It remains unclear what barriers might persist after such program implementation. This study explores residents’ and physicians’ perceptions of physical examinations teaching at the bedside following the implementation of a new structured bedside curriculum: What are the potentially persisting barriers and proposed solutions for improvement?

**Methods:**

The study used a constructivist approach using a qualitative inductive thematic analysis that was oriented to construct an understanding of the barriers and facilitators of physical examination teaching in the context of a new bedside curriculum. Participants took part in individual interviews and subsequently focus groups. Transcripts were coded and themes were identified.

**Results:**

Data analyses yielded three main themes: (1) *the culture of teaching physical examination at the bedside* is shaped and threatened by the lack of hospital support, physicians’ motivation and expertise, residents’ attitudes and dependence on technology, (2) *the hospital environment makes bedside teaching difficult* because of its chaotic nature, time constraints and conflicting responsibilities, and finally (3) *structured physical examination curricula create missed opportunities* in being restrictive and pose difficulties in identifying patients with findings.

**Conclusions:**

Despite the implementation of a structured bedside curriculum for physical examination teaching, our study suggests that cultural, environmental and curriculum-related barriers remain important issues to be addressed. Institutions wishing to develop and implement similar bedside curricula should prioritize recruitment of expert clinical teachers, recognizing their time and efforts. Teaching should be delivered in a protected environment, away from clinical duties, and with patients with real findings. Physicians must value teaching and learning of physical examination skills, with multiple hands-on opportunities for direct role modeling, coaching, observation and deliberate practice. Ideally, clinical teachers should master the art of combining both patient care and educational activities.

**Electronic supplementary material:**

The online version of this article (10.1186/s12909-018-1403-z) contains supplementary material, which is available to authorized users.

## Background

Throughout the mentored residency years, physicians in training must achieve a high degree of proficiency in performing complete and targeted physical examinations. This appears to be especially important in the management of complex patients with undifferentiated and multi-system diseases. The history and physical examination alone can accurately diagnose 60% of all admitted patients to Medicine, while repeated examinations on recently admitted patients, change diagnoses and management plans in more than one in four patients [1, 2]. Failure to adequately examine patients has the potential to promote faulty data gatherings, delays, and premature closures, which are commonly associated with medical errors [3].

Concerns are emerging about physicians’ abilities to rely on basic skills for clinical decision-making. Self-confidence and perceived value of performing physical exam maneuvers only slightly increase beyond the third year of medical school [4]. Students start cutting short elements of the physical examination upon completion of clerkship [5]. By the time they reach residency, not only do they bypass components of the exam, they also examine over hospital gowns as an acceptable equivalent and take less than 6 min to do so [6]. The perceived lack of value of physical examination skills may be due to time pressure faced by physicians, the diverging opinions clinical supervisors have on its utility across specialties, the role modeling they provide in performing less than exemplary examinations, and the lack of direct observation of trainees in performing the skills [5]. In the hope of saving time, trainees and mentors favor the ordering of expensive investigations, including special tests and imaging studies [7]. It is known that the use of basic skills, such as history taking and physical examination, has the potential to decrease the ordering of tests and consequently lower costs [2, 8].

Learning at the bedside appears to be an ideal environment for the acquisition of physical examination skills. Multiple learning theories including social learning, behaviorism, constructivism and the cognitive apprenticeship model are most activated at the bedside and support the idea of learning in such an environment [9, 10]. Moreover, these learning theories are embedded within the concept of deliberate practice, which were previously described as the ideal model for physical examination skill acquisition at the graduate level [11]. In the end, trainees have reported learning at the bedside as stimulating, while attending physicians concur that physical examination skills and bedside rounds are essential to patient care [12, 13].

Although arguments for teaching skills at the bedside are clear, time allocated to bedside teaching has declined considerably from 75% in the 1960s, to just 16% by the 1990s [14, 15]. A study looking at how post-graduate internal medicine trainees, or commonly called residents, spend their time during a shift revealed that only 12% of the time is spent at the bedside [16]. In recent years, several studies focused on identifying the barriers and possible solutions to bedside teaching in general. Those barriers were typically attributed to problems arising from clinical teachers, learners, patients, the culture shift and the workplace environment [17–19]. Strategies proposed included the need to create structured bedside curricula, have clear expectations for bedside rounds, and implement faculty development workshops [17, 19]. The previous implementation of skills improvement programs, targeted at teaching physical examinations, was in fact successful at increasing the frequency of both performing and teaching physical examination at the bedside [20]. It remains unclear what barriers might persist following the implementation of structured bedside curricula, specifically for the teaching of physical examination skills.

Therefore, this study explores residents’ and physicians’ perceptions of the bedside teaching of physical examination following the implementation of a new structured bedside curriculum: What are the potentially persisting barriers and proposed solutions for improvement?

## Methods

This study used a constructivist approach using a qualitative inductive thematic analysis that was oriented to construct an understanding of the barriers and facilitators of physical examination teaching in the context of a new bedside curriculum [21]. This approach was used to describe and understand barriers and facilitators through the coding and interpretation of data to identify themes. Participants were asked to participate in an initial 30-min individual interview and a subsequent one-hour homogenous focus group interview of residents or attending physicians. Written informed consent was obtained from all participants and the study was approved by the Ottawa Health Science Network Research Ethics Board (OHSN-REB).

### Setting

Prior to September 2013, bedside teaching was a scheduled one-hour weekly session for both junior and senior residents rotating through the clinical teaching units of the Ottawa Hospital, Canada. Each session was organized by one of the attending physicians on service for the week and happened on a voluntary basis. In our setting, the attending physician refers to a licensed physician in independent practice in charge of the inpatient service of the hospital. Physicians were given the freedom to choose the patient to be examined and the topic to be covered. There was no formal way of scheduling available teachers and keeping track of the topics covered. The sessions were commonly cancelled because of time constraints and lack of set expectations. The topics were repetitive as physicians would inadvertently choose to teach the same physical examinations. Trainees started demanding formal and consistent teaching, and attending physicians were noticing gaps in knowledge and skills. Consequently, in September 2013, a bedside teaching committee was formed and a weekly formal bedside teaching curriculum of physical examination skills was developed and implemented.

The curriculum is intended for the General Internal Medicine residents, post-graduate year 1 to 5 (PGY 1–5) studying at the University of Ottawa. The structured curriculum includes ten one-hour sessions covering various physical examinations, which are thought to be valuable to a general internist’s practice (Table 1). These topics were chosen and agreed upon following the distribution of a survey to all attending physicians within the Division of General Internal Medicine of our hospital, with a 50% response rate. Due to time constraint, residents were not consulted. The rounds take place at the bedside, with an admitted patient, and are led by the attending physician on the consult service for the week. The weekly topics are distributed to all involved to allow for preparation. The attending physician is responsible to find suitable patients with clinical findings that match the topic to be covered for the week. Attending physicians are also in charge of obtaining patient’s consent and can structure the session the way they like. No formal structure is suggested, although they are strongly encouraged to give direct feedback to the trainees involved and to add their own clinical pearls. Through formal weekly scheduling, expectations were set as both trainees and attending physicians were held accountable to attend and lead the sessions while respecting the pre-determined objectives. A tutor guide was developed and distributed ahead of time to provide guidance about the content of each session (see Additional file 1: Appendix 1).

**Table 1:** Bedside Teaching Curriculum of Physical Examinations

General Medicine Ward (At the bedside)		
Session	Specialty	Scenario
1	Cardiology	Blood pressure, JVP^a^, Ankle-brachial index
2	Cardiology	Vascular exam (AAA^b^, PVD^c^)
3	Respirology	Lungs landmark
		Clubbing
4	Respirology	Air flow limitation (COPD^d^)
5	Gastroenterology	Liver
		Spleen
		Ascites and chronic liver disease
6	Neurology	Fundoscopic exam
		Cranial nerves
7	Neurology	UMN^e^ vs LMN^f^
8	Endocrinology	Hypo/hyperthyroidism
		Thyroid gland (nodule)
9	Rheumatology	Shoulder
		Knee
10	Other	Deep vein thrombosis

Note

^a^Jugular venous pressure, ^b^ abdominal aortic aneurysm, ^c^ peripheral vascular disease, ^d^ chronic obstructive pulmonary disease, ^e^ upper motor neuron, ^f^ lower motor neuron

### Participants and data collection

All core residents and fellows in the Internal Medicine Residency/Fellowship Program at the University of Ottawa (*n* = 86) were invited to participate in this study via email and in person through an announcement at their academic half-day one year after the implementation of the curriculum. All attending physicians (*n* = 34) in the Division of General Internal Medicine were invited in the same way by the head of the division. Following informed consent, participants were individually interviewed by a trained non-biased research assistant for 30 min (for interview scheme, see Additional file 1: Appendix 2). Subsequently, participants who had completed the structured interviews were asked to participate in a homogenous one-hour focus group interview of residents or attending physicians (for interview scheme, see Additional file 1: Appendix 3). Data collection took place between August and October 2014, while the new curriculum was initially implemented in September 2013. All participants were exposed to at four sessions of the new curriculum.

The initial interview process relied on an approach incorporating open-ended questions such as “What is your experience with bedside teaching in general? Tell me more?” and moved towards asking directed questions such as “What are some of the barriers encountered during the physical examination bedside teaching sessions?” This was done to help the researchers understand the lived phenomenon, but also to help them construct a follow up interview scheme which would be used during the focus group interviews.

### Data analysis

Our research team consisted of three researchers, all of whom held formal training and experience in qualitative methods. The interviews and focus group interviews were digitally audio-recorded, transcribed *verbatim* and anonymized prior to data analysis. No personal identification data of participants were collected.

Through an inductive and iterative process, data was examined, coded and organized into concepts, categories and themes by two of the researchers (M.R. and C.T.). The researchers met after individually coding the initial six interviews and reached consensus on an initial coding structure and emerging themes [22]. Based on these emerging themes, the interview scheme for the focus group interviews was developed. Further interviews were analyzed until saturation was reached and final themes were agreed upon. Once data analysis was completed, an email, describing the findings of the study, was sent to all participants. Member checking with the attending physicians and residents was conducted allowing the participants to comment on the accuracy of the results of the qualitative analysis. Data analysis was done with the help of a qualitative computer software (Nvivo for Mac, 2014, Qualitative Research Software-QRS International). Trustworthiness of results was reached through triangulation using two sets of data collection (semi-structured interviews and focus group interviews), by having two of the authors participate in the iterative data analysis, and by conducting member checking.

## Results

Nine internal medicine residents (one first year, seven second year, and one fifth year) and twelve attending physicians with one to twenty years of experience volunteered to participate in the interviews. Five residents and five attending physicians participated in the focus group interviews. All participants were exposed to the formal bedside teaching rounds of physical examinations. Several perceived barriers and solutions related to bedside teaching were highlighted from all participants and were coded into several concepts, categories and ultimately themes.

Data analysis yielded three main themes: (1) the culture of teaching physical examination at the bedside, (2) the hospital environment makes bedside teaching difficult, and finally (3) structured physical examination curricula create missed opportunities. All concepts pertaining to each of these themes were further organized into two major sections: the barriers and the suggestions for improving the bedside teaching of physical examinations to improve clarity (Table 2).

**Table 2:** Themes of the barriers and suggestions to improve the teaching of physical examinations at the bedside

Main Themes	Categories	Barriers	Suggestions for improvement
The culture of teaching physical examination at the bedside	Hospital	● Lack of standard/motivation ● Inadequate appreciation, award, and salary support	● Need to set standard ● Increase appreciation, award, and salary support
	Attending physicians	● Lack of enthusiasm ● Lack of physician confidence ● Impact of prior education and exposure ● Different knowledge, attitudes and skills among physicians ● Lack of accountability	● Need to work on the impact of prior education (Increasing need to train residents as teachers) ● Improve teachers selection and preparation ● Create faculty development programs
	Residents	● Lack of enthusiasm and motivation ● High anxiety/stress level ● Poor insight in their own skills	
	Technology	● Over reliance on technology	
The hospital environment makes bedside teaching difficult	Time pressure	● Conflicting responsibility ● Too many admitted patients	● Prioritization of teaching (too many teaching rounds) ● Delegation of task
	Hospital Environment	● Rooms are too small ● Lack of basic equipment ● Too noisy ● Need for nurse education regarding paging and protected time	● Purchase of basic equipment for examination ● Utility of dedicated room for teaching ● Need for nurse education regarding paging and protected time ● Need for geographic location for each team
	Patients	● Not available ● Fear of discomfort ● Family disruption	● Importance of proper patient selection
Structured physical examination curricula create missed opportunities	Content	● Missed opportunity by following curriculum (restrictive) ● Teachers not following the curriculum / schedule ● Inability to find patient with findings	● Importance of proper patient selection ● Incorporation of ultrasound, media, video, photos into rounds
	Organization	● Lack of schedule dissemination ● Need for formal structure of the rounds, debrief session	● Need to give topic ahead of time for residents to prepare
	Assessment	● Lack of assessment tools	● Implement mini-CEX or encounters cards to promote formative feedback

### The culture of teaching the physical examination at the bedside

The culture of teaching the physical examination at the bedside was found to be shaped and threatened by the lack of hospital support, physicians’ motivation and expertise, residents’ attitudes and dependence on technology**.**

#### Barriers

Participants highlighted the overall lack of recognition and expectation at the divisional, departmental and hospital wide level to teach physical examination at the bedside. They also referred to the new generation of graduating physicians who now lacks knowledge in teaching a skill which they never fully learned themselves.

- “So I think it does start there with some of the higher ups acknowledging that teaching is important.” (Attending physician 7)

They stressed the importance of attending physicians’ attitude and their lack of dedication and accountability towards teaching at the bedside. Physicians admitted their own lack of expertise and need for incentive.

- “I went to the sessions four times and my experience was very different from one preceptor to another. One physician that gave us a session was very precise, gave us a lot of good information and a lot of new stuff that I hadn’t learned before. Whereas another physician, it was a lot more general.” (Resident 1)
- “I think the degree of commitment to the quality of the bedside teaching is staff dependent.” (Attending physician 10)

Challenges perceived by and for residents were linked to their attitude and dedication toward the teaching. This included their anxiety to perform in front of peers, their lack of motivation, knowledge and insight into their own skills. They disliked being the focus of attention and wished they could prepare before each session.

- “It’s something that can be a bit stressful and make you feel a bit vulnerable because you’re there with your peers. You’re being put on the spot.” (Resident 4)
- “I think it’s a more… it’s a richer experience if you have a base knowledge of what you’re about to do and that kind of everybody in the group has a base knowledge.” (Resident 1)

Finally, it appears that the movement toward technology and away from the bedside caused physical examination skills to be lost.

- “You know, it is hard because we are trying to buck a trend moving away from clinical medicine towards imaging, imaging, imaging and very abbreviated examination because the proof apparently is not there.” (Focus group, attending physician M2)

### Suggestions for improvement

Under the same theme, several comments emerged in regards to possible strategies to break down cultural barriers. Recognizing that physical examination is embodied in the art of doctoring would represent a step forward. This would help value that the time spent at the bedside is essential for thorough assessments of complex patients. Setting up more standards and expectation for both the attending physicians and residents beyond the structured curriculum was proposed. It was also thought that elaborating a clear intent for bedside teaching sessions may make them more likely to occur by holding all parties accountable. Other suggestions to change the culture were toward emphasizing the need to train residents as bedside teachers during residency.

- “So I think we, as staff people, we have to take a concerted effort and recognize that teaching of physical examination and really the art of doctoring is actually really important. So I think we have to change the attitude for us and same thing with medical students and residents. We need to impart upon them that doing a physical exam, like taking history and a physical really is important still, even in the 21st century. If you don’t have that foundation, you really don’t have anything but a house of cards.” (Attending physician 6)
- “So you could teach most days but almost every day is something you could aim for at least. I think you set that as a standard.” (Attending physician 7)

Residents suggested that a better selection process and preparation of the attending physicians involved in teaching would help improve the enthusiasm and the quality of the sessions. Attending physicians exclusively proposed that a dedicated faculty development workshop would help build their confidence and knowledge of specific skills and techniques and that an incentive or award program would encourage participation.

- “I think if we had refreshers for staff physicians that might also be helpful. The same way that we have like ACLS refreshers and those types of things, which we always find embarrassing when we go because those are things that we should know. I think it helps partly the preceptors’ confidence and I think might help promote sort of the number of physical exams sessions that we do.” (Attending physician 9)
- “I think that there should be a more standardized approach perhaps. I think some of the staffs are good at identifying like an issue that you’re examining for or doing it in a Royal College format. And then other staffs are just way off on a tangent on like random stuff and just… like with no focus or just things that they think are interesting that don’t really seem to go together or don’t seem to be answering the question.” (Focus group, resident F3)
- “One way to try and emphasize this it to have an award. You know, we have, Extra Mile Award. Maybe we should have the, you know, the Beautiful Physical Diagnosis of the Month Award.” (Focus group, attending physician M2)

### The hospital environment makes bedside teaching difficult

This theme illustrates that the hospital environment makes bedside teaching difficult because of its chaotic nature, time pressures and conflicting responsibilities.

#### Barriers

The most commonly identified barriers from the environment referred to the business of the medicine service from the large number of admitted patients on the ward and its unpredictability when caring for sick patients. Both residents and attending physicians stressed the impact of time pressure and conflicting responsibilities. Attendings reported the constant rise in clinical duties when working on the hospital ward and the competing interest of their parallel outpatient practice and administrative duties. Residents mentioned the difficulty in juggling multiple obligations of looking after patients, being on-call, or at lectures and academic half-days.

- “The way that internal medicine has changed now, it’s so busy and so many responsibilities, so many expectations. Really the turnaround has to be fast. There is no engagement with bedside teaching. We do have protected time for bedside teaching, it’s almost impossible to get the juniors or the residents to go. They are late. You have to be paging them. They always have to leave. It’s just part of the system, right.” (Attending physician 3)
- “(A barrier) for me was always the workload because it is a very busy service and there’s always this expectation that things get done… You’re going to be stressed to just try and get things done rather than trying to learn.” (Resident 7)

The crowded patients’ rooms, the lack of easily accessible basic equipment for examination and the constant pager interruption from nurses and other co-workers were also mentioned.

- “It is hard to get a lot of the equipment so you can’t get an ophthalmoscope. You can’t get an otoscope. Sometimes you can’t get a reflex hammer. So I have to come back down to my office and get stuff.” (Attending physician 11)
- “So I think from a sort of logistical point of view, like obviously if you’re in a four-patient room and you come in with a team of 10 people, that’s a little bit difficult.” (Attending physician 10)

In regards to patients, participants expressed concerns about the need to choose the right patient, about their overall availability, the fear of causing discomfort and the disruption caused by family members. Patients were often gone for tests, in isolation rooms or too ill to participate in teaching.

- “When you do get to the bedside it’s all about other stuff. Like the patient’s away for the test. They’re on the bedpan. They’re in a bad mood. They’re saying no.” (Attending physician 12)

#### Suggestions for improvement

Participants proposed solutions in highlighting that the work environment could easily benefit from the purchase of further physical examination tools and that the use of a dedicated room for teaching would be of value.

- “The department should be able to supply some of the equipment, kind of like a little suitcase for your senior residents” (Focus group, attending physician F3)

Participants emphasized the importance of selecting the right patients for teaching. They proposed dissemination of patients’ names with interesting clinical findings among members of divisions. Most participants agreed that teaching with a patient with findings was extremely valuable to put the topic into context.

- “Getting a good patient is half the battle and maybe if we disseminate names in on our division, we’re looking for a spleen this week. Any good patients?” (Focus group, attending physician F2)

Finally, very few solutions were proposed to mitigate time constraints. In general, attending physicians and residents agree that if there was a way to delegate some of the administrative duties and to decrease the volume of educational events in any given week, this could be of benefit.

- “In terms of not having enough time, obviously some of these ridiculous administrative things that we’re having to do need to be delegated to more appropriate people so that we actually have more time in the day.” (Attending physician 11)

### Structured physical examination curricula create missed opportunities

This theme notes the missed opportunities of the structured physical examination curriculum. It was found to be restrictive and posed difficulties in identifying patients with findings.

#### Barriers

The current formal bedside curriculum blueprint was not considered to be representative of the practice of a general internist. Several participants believed that certain physical examination skills being taught were rarely or never used in general medicine, such as the ankle-brachial index. It was also perceived as being somewhat restrictive, in the sense that the physical examination skills scheduled were not in line with the interesting pathology of admitted patients on a given week. As well, a few teachers did not follow the pre-determined schedule leading to confusion, and repetition of topics from one week to the next.

- “As mentioned before, some of what we’re being asked to teach is perhaps a little bit foreign to us because it’s just not part of what we do as routine as general internists.” (Attending physician 1)
- “Sometimes it’s an issue of expertise. I certainly feel uncomfortable, and I know I do a very poor job, when I’m teaching something that is not part of what I was ever taught, you know, to do, an ankle brachial…” (Focus group, attending physician M1)

#### Suggestions for improvement

In terms of solutions, clinicians suggested removing skills and maneuvers of limited use from the curriculum. Residents proposed to distribute the topic of each session in advance and suggested incorporating technology in bedside teaching sessions, such as ultrasound, videos and photos. Finally, the implementation of assessment methods for both clinical teachers and residents was proposed to be crucial in ensuring quality assurance, with perhaps the use of mini-CEX or encounters cards to promote formative feedback. A few residents also suggested summative assessment as a tool to drive learning.

- “I think that we would do better if we had a chance to go over what we are supposed to teach and say this is relevant, this is reasonable, this is real like, this is exam and this is not. Get rid of the stuff that is not relevant to us.” (Focus group, attending Physician M1)
- “So not that learning physical exam is useless but certainly it would be more imminent if you tell them that they have a quiz in a couple of days.” (Resident 7)

## Discussion

Despite strategies to increase the frequency and quality of physical examination teaching at the bedside, such as the implementation of weekly formal physical examination rounds and the creation of a tutor guide, our study suggests that several challenges remain at play. Although the findings of this study resonate with earlier evidence, in which commonly identified barriers to bedside teaching related to attending physicians, learners, patients, time and system issues [17], the current study provides deeper insights into the barriers and solutions in the context of a structured bedside curriculum. Previous evidence studied bedside teaching in the general sense, where teaching is integrated into patient care and delivered without pre-determined learning objectives.

While reflecting on the challenges and solutions proposed by participants in this study, several areas were identified that may improve the implementation of structured bedside teaching curricula moving forward. Overall, the biggest barriers were attributed to the impact of culture on bedside teaching, the hospital environment and the restrictions caused by the curriculum. Consequently, avenues opened to improvement include (1) fostering a culture change and (2) adjusting the existing structured curriculum.

### Fostering a culture change

Setting up standards and expectations for bedside rounds appears to be an ongoing battle. Hospital administrators and educators must recognize that academic physicians have a unique role in delivering patient care and teaching residents. While history taking and physical examination requires a minimum amount of time, the same hold true for delivering teaching rounds. This concept of minimal set-time to perform a task was highlighted by Cohn [23] in his analogy describing that a maximum number of patients could undergo surgery on any given day. For the same reason, a maximum number of patients should be admitted under one attending physician at any given time to allow for both patient care and educational activities to take place.

Higher numbers of admitted patients per team lead to time pressure, increased responsibilities and length of stay and cost [24]. Prior recommendation regarding the optimal inpatient workload for US hospitalists ranged from 10 to 15 patient visits per day [24]. Similarly, prior studies suggested that setting up a census cap of 14 admitted patients improved residents’ perceptions of workload, conference attendance, duty hours compliance, and 30 day patients’ readmissions rates [25]. In a recent national survey of hospitalists, respondents reported that increased workload leads to delays in care, poor communication between physicians and patients, the ordering of unnecessary tests, and complications [26]. Further research investigating the ideal patient census in academic hospitals which offers a balance between learning opportunities and optimal patient care should be pursued.

Helping physicians master the art of combining both patient-care and teaching rounds at the bedside through appropriate targeted faculty development could be another suggestion to facilitate the teaching of physical examinations and foster a culture change. The advantages of learning in a clinical context are clear and structured rounds might not need to be a separate event. Instead, residents may benefit from having teaching imbedded within the clinical delivery of care. Prior studies have shown that only 7–12 min is required per patient during rounds which include reviewing history, performing a targeted physical examination, discussing care and placing orders [27, 28]. While ward rounding improves quality of education, it also increases patients’ satisfaction, while decreasing medical errors, mortality rates and lengths of stay [27, 28]. It might also address the difficulty of identifying patients with findings. In the end, initiatives targeted at helping physicians gain insights on practical methods to effectively implement patient-centered ward rounding that is time efficient and integrates teaching should be prioritized. In the meantime, at local level, other ways to emphasize the value of bedside teaching lie in the development of recognition programs and faculty development days to support physicians.

### Adjusting the existing structured curriculum

The current study further highlights important take-home messages in regard to the development of a structured bedside curriculum. Physical examination bedside rounds require a high level of physicians’ expertise in specific skills and techniques. Several concerns emerged from attending physicians and residents about the complexity and quality of the formal rounds. Attending physicians believed that some topics were challenging for them to teach, as some bedside examination skills were not commonly encountered in their daily practice. There thus seems to be a disconnect between the skills internal medicine physicians are expected to be competent in performing as role-models, and the skills are needed and used on a daily basis to care for patients. This disconnect may have led to the loss of some physical examination skills which are no longer commonly utilized in practice, perhaps as a consequence of the ongoing advancement in the field of diagnostic imaging. Consequently, new skills used in the assessment of the physical examination, such as the use of ultrasonography, must find their way into such a curriculum to stay current. Offering an opportunity for attending physicians to refresh, refine and update their physical examination skills and/or involve a selected group of outstanding teachers for the formal rounds are options to be explored. In this study, concerns also emerged regarding residents’ lack of motivation to improve their own physical examination skills. Further studies should aim to better understand the reason behind this disinterest. Studying positive strategies to drive learning, such as whether the implementation of frequent in-training physical examination assessments improves attitudes and outcomes, should also be part of future research.

Moreover, a structured curriculum may be perceived as restrictive. Finding the balance between organizing teaching physical examination with patients with interesting findings and ensuring that all the skills and techniques are covered is a challenge for educators. Teaching a skill with a patient who has no findings may lead to major disengagement from both teachers and learners. The principal goal of a structured curriculum is to ensure that basic topics are covered and that residents can prepare ahead of time by having the topics distributed in advance. A solution could be to compromise: while a specific important skill or technique could be covered in half a session, there should be some flexibility within the allocated time to allow for an interesting and available case to be reviewed.

Moreover, effort to improve communication across attending physicians needs to become a priority in helping identify appropriate patients. Ideally, hospital software would allow physicians to flag admitted patients with interesting findings for teaching purposes. Although prior literature suggests that avoiding patients with limited verbal skills, difficult interaction styles, infectious or acute diseases would facilitate teaching, they might still represent value in learning from difficult scenarios [29]. Another avenue to mitigate this problem would be to more effectively incorporate technology into the teaching. It might be interesting to look at developing banks of videos and pictures to facilitate teaching. Finally, as mentioned above, incorporating the physical examination rounds within patient care rounds might help alleviate the problem of identifying patients with findings.

## Limitations

This study was carried out at a single institution and thus the barriers and solutions may be only locally applicable. The results of our study are furthermore limited by the fact that enrollment was voluntary and that response rates were only 10% (9/86) for residents, and 35% (12/34) for attending physicians. Therefore, the opinions gathered of the attending physicians may be those of interested educators rather than representatives of the entire group, although including more than 30% of the attending physicians may have minimized this. Attending physicians and residents were interviewed separately, which implies that they were not able to reply to each other’s comments. Another study bringing different stakeholders together in a focus group interview would be interesting.

## Conclusions

Despite the implementation of a structured bedside curriculum for physical examination teaching, our study suggests that cultural, environmental and curriculum-related barriers remain important issues to be addressed. Institutions wishing to develop and implement similar bedside curricula should prioritize recruitment of expert clinical teachers, recognizing their time and efforts. Teaching should be delivered in a protected environment, away from clinical duties, and with patients with real findings. Physicians must value teaching and learning of physical examination skills, with multiple hands-on opportunities for direct role modeling, coaching, observation and deliberate practice. Ideally, clinical teachers should master the art of combining both patient care and educational activities.

## Practice Points

1. Physicians and health care institutions need to emphasize the value of learning in the sociocultural context allowing for direct role modeling, coaching and observation which is essential for complex skill acquisitions.
2. Physicians must be supported through faculty development workshops to develop their bedside teaching and physical examination skills.
3. Physicians should master the art of combining both patient care and educational activities for better time management.

## Definition of terms

1. Structured bedside curriculum: A planned and scheduled curriculum, which has predetermined learning objectives.
2. Resident: A post-graduate medical education trainee
3. Attending physician: A licensed physician in independent practice in charge of the inpatient service of the hospital.

## Additional file

Additional file 1: Appendix 1. Physical Examination Bedside Teaching Guide. Appendix 2. Semi-structured individual interview questions and prompts. Appendix 3. Focus group interview questions and prompts. (DOC 4288 kb)

## Abbreviations

AAA: Abdominal aortic aneurysm
COPD: Chronic obstructive pulmonary disease
JVP: Jugular venous pressure
LMN: Lower motor neuron
M.R. and C.T: Researchers Melissa Rousseau and Claire Touchie
Mac: Macintosh computer
Mini-CEX: Mini-Clinical evaluation exercise
OHSN-REB: The Ottawa Health Science Network Research Ethics Board
PGY: Post graduate year
PVD: Peripheral vascular disease
QRS International: Qualitative Research Software International
UMN: Upper motor neuron
US: United States
